# Tracking maternal, infant, and young child nutrition in Brazil after a decade without evidence

**DOI:** 10.1590/0102-311XEN116023

**Published:** 2023-10-23

**Authors:** Paulo Augusto Neves

**Affiliations:** 1 Centre for Global Child Health, The Hospital for Sick Children, Toronto, Canada.

Monitoring and tracking the health and nutrition of populations are essential parts of the global commitment to improve overall well-being ^1^ ^,^ ^2^. The United Nations 2030 Agenda for Sustainable Development Goals (SDG) acknowledges the importance of solid and robust data availability at national and regional levels for monitoring the progress and policy across nations ^3^. Nationally-representative household surveys have been conducted worldwide, especially in low-resource settings, and helped to understand the achievements and challenges that countries must overcome to reduce the burden of illnesses according to each context. Considering that health inequalities persist worldwide, this type of data also allows for the investigation of disaggregate estimates by socioeconomic level, education, geography, among others. The path towards narrowing such inequalities depends on the production of evidence to track the status of minorities, within and between countries, and, also, to support practices, programs, and policies to reduce the gaps and to trace the impact of intervention ^2^. Such evidence could guide health and well-being policies in a better cost-effective way ^4^.

In Brazil, a large country with the largest economy in Latin America, a lack of evidence on maternal-child nutrition indicators remained for 13 years until the completion of the 2019 *Brazilian National Survey and Child Nutrition* (ENANI-2019). In this sense, most public policies and pragmatic activities targeting mothers and child nutrition relied upon the results of the 2006 *Brazilian National Survey on Demography and Health of Women and Children* (PNDS 2006) ^5^. Castro et al. ^6^ reported the findings of descriptive trend analysis of child nutrition indicators of international relevance comparing both surveys on domains such as anthropometry, feeding practices, and micronutrient deficiencies. Despite improvements being noted on some indicators during the period, others did not change, and some even worsened.

Over decades, Brazil had been classified as a country where anemia and vitamin A deficiency were moderate public health problems, especially affecting the most disadvantaged in the poorest areas. The ENANI-2019 showed that important progress in the reduction of both micronutrient deficiencies was accomplished since they are now classified as mild public health problems with striking reductions in the inequalities by region, maternal education, and race/skin color ^7^ ^,^ ^8^. The ENANI-2019 included recommended and reliable methods of collection, transportation, storage, and analysis in the micronutrient assessment, which increased the reliability of results, whereas the PNDS 2006 presented methodological concerns that could have negatively affected their results (for example, ENANI-2019 used capillary blood sample for the retinol essays, whereas the PNDS 2006 used dry blood spot). Despite the good news, current policies in Brazil require evaluations to deal with anemia and vitamin A deficiencies. These evaluations should be based on the current epidemiological scenario and the new scientific evidence that enacts the importance of supplementation with multiple micronutrients at low doses by food fortification (as used in the Brazilian National Strategy for Fortification of Infant Feeding with Micronutrients in Powder - NutriSUS ^9^) and antenatal care supplementation ^10^.

Regarding breastfeeding indicators, despite the non-significant increase in the prevalence over time, it still lags behind international targets for the promotion of optimal breastfeeding practices, such as the World Health Assembly target of at least 70% of children under 6 months of age being exclusively breastfed by 2030. However, advances were reported in the reduction of inequalities, such as the gap between regions, education levels, and race/skin color. Brazil shows a unique and incredible trajectory regarding breastfeeding promotion, including the successful experience with human milk banks and proper work leave to women after childbirth ^11^ ^,^ ^12^. Although the exemplary country’s trajectory, the findings from Castro et al. ^6^ indicate that pro-breastfeeding efforts and actions must be intensified to support, promote, and protect breastfeeding. Special attention should be given to the increasing rates of formula in children’s diet ^13^ and C-section ^14^ in Brazil, which are known factors that hamper optimal breastfeeding practices.

The findings for child anthropometric indicators are intriguing. Over the last decade, no progress has been documented regarding the reduction of malnutrition among children under 5 years old in Brazil ^6^. Castro et al. ^6^ observed an upward trend in the prevalence of overweight that almost doubled during the period, with the highest increases documented in the wealthiest regions and among people who self-declared as white. This trend is associated with unhealthy lifestyle habits, such as less physical activity and increasing consumption of unhealthy foods. Additionally, the nutritional status of mothers can help explain the results for children, whereas the parent’s and offspring’s body mass index (BMI) are directly correlated; therefore, family history of unhealthy habits could be investigated. The prevalence of stunting has not changed in the last 13 years (around 7%), even though inequalities were reduced, especially among regions. However, the stunting increased only among children under 24 months of age, showing that different risk factors might act according to age on the occurrence of stunting in children under 5 years old. Considering the window of opportunity of the first 1,000 days of life ^15^, pregnancy-related and early life factors must be considered as potential approaches to reduce the burden of stunting among young Brazilian children.

Previous Brazilian household surveys have not collected the necessary information to generate internationally relevant child feeding practices indicators. The ENANI-2019, brought attention to the topic on the newly recommended World Health Organization/United Nations Children’s Fund (WHO/UNICEF) feeding indicators by reporting the high prevalence of children who consumed ultra-processed foods, along with the fact that a quarter of children did not consume any fruits and vegetables in the day previous to the survey ^6^. Moreover, most children consumed animal-based protein-rich foods, whereas nearly 40% still did not achieve a minimum dietary diversity. These findings highlighted the challenges that policies and programs must overcome based on the current situation, aiming at targeting the predatory marketing of ultra-processed foods for children. Meanwhile, the promotion of healthy eating habits must be revised to increase the consumption of fresh foods. Lastly, the figures ^6^ were estimated for children 6-59 months of age, whereas estimates for children 6-23 months of age are necessary for the assessment of complementary feeding practices in Brazil and for international comparisons.

ENANI-2019 also shows remarkable improvements in demographic characteristics that are directly associated with the indicators studied in the analysis. The changes over time reported by Castro et al. ^6^ are partially due to the improvements in the living conditions of the population. It is important to note the increase in the number of households with access to pipped water, sewage system, and internet access, as well as the increase in mothers’ schooling level. Despite these positive results, improvements are still possible. About 25% of the households lack access to general sewage, and 22% of mothers have under 7 years of education. Findings by region would help to understand where more investments and actions are necessary, but, presumably, the poorest areas in the North and Northeast, and slums of big metropolitan areas still suffer the most with poor living conditions. Generally, some of the challenges for improving nutrition indicators in Brazil, such as the reduction of stunting, are tightly bounded to improvements in the living conditions in the future.

In conclusion, while old public health and nutrition problems must be closely tracked, including the prevalence of stunting and micronutrient deficiencies, new challenges emerge as society progresses, such as the increasing prevalence of overweight and the high consumption of ultra-processed foods among Brazilian children. This epidemiological scenario highlights the importance of health policies based on solid findings. The ENANI-2019 filled the 13 years gap in the evidence on maternal-child nutrition in Brazil and allowed cross-country comparisons of children’s nutritional status as part of international efforts to promote health and well-being of societies, such as the SDG. Monitoring novel public health problems that threaten past achievements , including the COVID-19 pandemic, is imperative and urges the necessity of standardized data periodically produced. A new edition of the ENANI is planned for 2024 and will help monitor the advances made in Brazil for reducing the burden of malnutrition and promoting optimal feeding practices among children under 5 years old.
